# Study on the Thermal Distribution Characteristics of a Molten Quartz Ceramic Surface under Quartz Lamp Radiation

**DOI:** 10.3390/mi14061231

**Published:** 2023-06-11

**Authors:** Hao Chen, Wei Li, Shimin Zhu, Aiqiang Hou, Tao Liu, Jiangshan Xu, Xiaowei Zhang, Zao Yi, Yougen Yi, Bo Dai

**Affiliations:** 1 The State Key Laboratory of Environment-Friendly Energy Materials, School of Materials and Chemistry, School of Information Engineering, School of Mathematics and Science, Southwest University of Science and Technology, Mianyang 621010, China; chenhaoswustedu@163.com (H.C.); 13350956494@163.com (W.L.); 18780550401@163.com (S.Z.); godloveqiangge@outlook.com (A.H.); liut427619@163.com (T.L.); xvjiang121@163.com (J.X.); xiaoweizhang@swust.edu.cn (X.Z.); yizaomy@swust.edu.cn (Z.Y.); 2 School of Chemistry and Chemical Engineering, Jishou University, Jishou 416000, China; 3 College of Physics and Electronics, Central South University, Changsha 410083, China; yougenyi@csu.edu.cn

**Keywords:** molten quartz ceramics, quartz lamp heating, temperature uniformity, heat flow density

## Abstract

More and more researchers are studying the heat transfer performance of aeronautical materials at high temperatures. In this paper, we use a quartz lamp to irradiate fused quartz ceramic materials, and the sample surface temperature and heat flux distribution were obtained at a heating power of 45~150 kW. Furthermore, the heat transfer properties of the material were analyzed using a finite element method and the effect of surface heat flow on the internal temperature field was investigated. The results show that the fiber skeleton structure has a significant effect on the thermal insulation performance of fiber-reinforced fused quartz ceramics and the longitudinal heat transfer along the rod fiber skeleton is slower. As time passes, the surface temperature distribution tends to stability and reaches an equilibrium state. The surface temperature of fused quartz ceramic increases with the increase in the radiant heat flux of the quartz lamp array. When the input power is 5 kW, the maximum surface temperature of the sample can reach 1153 °C. However, the non-uniformity of the sample surface temperature also increases, reaching a maximum uncertainty of 12.28%. The research in this paper provides important theoretical guidance for the heat insulation design of ultra-high acoustic velocity aircraft.

## 1. Introduction

With the rapid development of aerospace and national defense industries, high-temperature wave-permeable ceramics have become important for key components such as antenna covers and radar antenna windows [1,2,3]. They are one of the current research focus areas in the field of materials [4,5,6,7]. The purpose of the high-temperature transmission material is to act as a multi-functional dielectric material for the protection of the communication, telemetry and guidance systems of aircraft under harsh environmental conditions. It is widely used in launch vehicles, spacecrafts, missiles and re-entry satellites [8,9,10,11,12]. With the continuous improvement in aircraft speed, the environment of heat and force loads to which aircraft are exposed during flight is becoming more and more severe. In addition, there are more stringent requirements for high-temperature permeable materials and structural shapes. Therefore, it is important to study the variation law of the surface heat distribution of high-temperature permeable materials under radiant heat [13,14,15]. The magnitude and direction of heat flux density can characterize the degree and direction of heat transfer, so the heat distribution on the surface of the sample can be described more accurately by using heat flux density, and the direct measurement of heat flux density by heat flux sensors is less affected by the external environment. Therefore, quantitative analysis of the influence of heat flow on the internal temperature field of the structure provides guidance for determining the heat transfer performance of high-temperature breathable materials under radiant heating and optimizing structural design. 

Molten quartz ceramic is a kind of homogeneous ceramic material that is produced from molten quartz powder or quartz ceramics as the raw material by means of crushing, molding, sintering and other ceramic manufacturing processes [16,17,18,19]. Jesse Maddren et al. established one-dimensional heat conduction for ceramic fiber thermal insulation material, and analyzed the influence of internal radiation thermal conductivity [20]. Wu Dafang et al. established the measured nonlinear relationship model between the thermal conductivity of lightweight high-temperature ceramic insulation and temperature and then finished a numerical simulation. The results show that the numerical calculations and test results achieved good consistency. The non-linear relationship between the measured thermal conductivity and temperature can provide accurate calculation results. To allow the theoretical calculation to replace, to some extent, expensive pneumatic thermal simulation testing requires a reliable foundation [21]. To clarify the relationship between thermal insulation performance and structure quality, Sun Lei compared and analyzed the temperature field around an aircraft at 0°, 2° and −2° flight attack angle, based on the typical multi-layer thermal insulation structure of hypersonic aircraft in adjacent space. He also analyzed the influence of the thickness change in different material layers on the thermal insulation performance [22]. In summary, in recent years, fused quartz ceramics, with their excellent ablation resistance, heat resistance, high-temperature mechanical properties and integrated forming technology have effectively met the requirements of hypersonic aircraft. However, research on the surface temperature uniformity and the accurate measurement of heat flux density of molten quartz ceramics in a radiant heating environment is not clear. Further research is needed [23,24].

In this paper, a high-power quartz lamp is used to heat the molten quartz ceramics to obtain the heat flux density value and surface temperature distribution of the irradiated sample. Test data are compared and the results analyzed. Then, we perform a finite element calculation to simplify the model under the same temperature boundary conditions of simulation and conduct quantitative analysis of the influence of heat flow on the internal temperature field. We can provide guidance on the fiber-reinforced molten quartz ceramic high-temperature wave material of heat transfer performance analysis and structure optimization design.

## 2. Structure and Design

**Figure 1:**
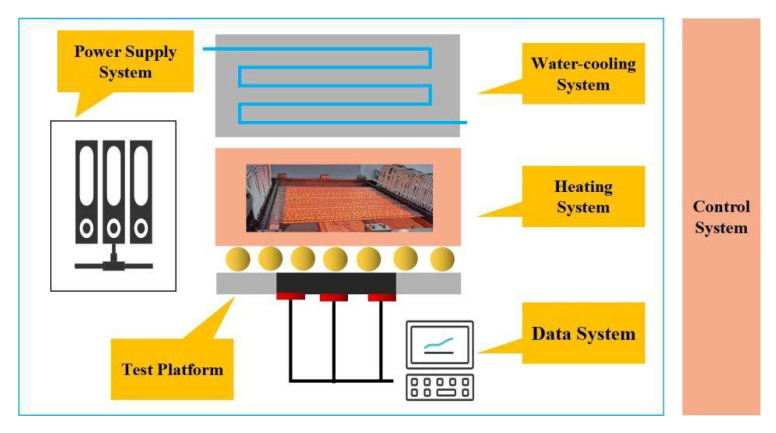
Schematic diagram of a quartz lamp radiation heating test system consisting mainly of a power supply system, a water cooling system, a heating system, a test platform and a data system. Diagram of quartz lamp radiation heating test system.

As a common radiation heating equipment in structural thermal testing, the quartz lamp array has the characteristics of small thermal inertia, easy control and strong adaptability to complex structures; thus, it is widely used in aircraft structure thermal testing [25,26,27]. The high-power infrared radiation long-time thermal assessment platform is composed of power supply system, test platform, water cooling system, heating system, data system and control system as shown in Figure 1. The quartz lamp array of the heating module consists of 30 quartz lamps arranged in parallel and side by side. The quartz lamp spacing is 2 cm. The quartz lamp filament heating length is 660 mm. The length of the quartz lamp tube is 760 mm. The maximum heating power is 150 kW and the heat flow density of the uniform area is about 160 kW/m^2^. The surface heat flux control accuracy of the specimen is less than 3 kW/m^2^. It can realize heterogeneous controllable heat flow load. The lamp heat flow is independently controllable; the control accuracy is better than 1 kW/m^2^ and a high-temperature environment is provided by means of radiant heating. During the experiment, the sample is first placed on the test platform. Then, the heating power of the quartz lamp is controlled by a temperature controller, which gradually warms up the sample. At the same time, a data collector records the temperature change in the sample and other relevant data.

A part of the data system is connected to a heat flow sensor, which collects the temperature distribution data of the lower surface of the sample. The heat flow sensor we use is mainly from our own ALHS thin-film heat flow sensor, which we have developed. Its main mechanism is the lateral Seebeck effect, so it has excellent characteristics of fast response. It has a maximum operating temperature of 300 °C, a response time of 30 μs and a heat flow range of 100–200 MW/m^2^. In addition, the system includes a K-type thermocouple to measure both heat flow and temperature. 

The temperature distribution was determined by solution of the energy conservation formula using the heat transfer analysis module of the Abaqus finite element software [28,29,30]. The formula for the three-dimensional heat transfer is as follows [31,32]:

(1) $$\rho c_{p}(T)\frac{\partial (T)}{\partial t}=k(T)\left(\frac{\partial^{2}\left(T\right)}{\partial x^{2}}+\frac{\partial^{2}\left(T\right)}{\partial y^{2}}+\frac{\partial^{2}\left(T\right)}{\partial z^{2}}\right)$$

In the formula: *ρ* represents the density of the object, in kg·m^−3^; c_p_ represents the specific heat capacity of the object, in J·(kg·°C)^−1^; k(*T*) represents the thermal conductivity of the object, in W·(m·°C)^−1^; *T* represents the temperature of the object, in °C; and *t* represents the thermal conduction time of the object, s.

Further derivation of Equation (1) yields:

(2) $$\frac{1}{\alpha }\frac{\partial (T)}{\partial t}=\left(\frac{\partial^{2}\left(T\right)}{\partial x^{2}}+\frac{\partial^{2}\left(T\right)}{\partial y^{2}}+\frac{\partial^{2}\left(T\right)}{\partial z^{2}}\right)$$

where: *α* = k/*ρc* indicates the thermal diffusivity.

**Figure 2:**
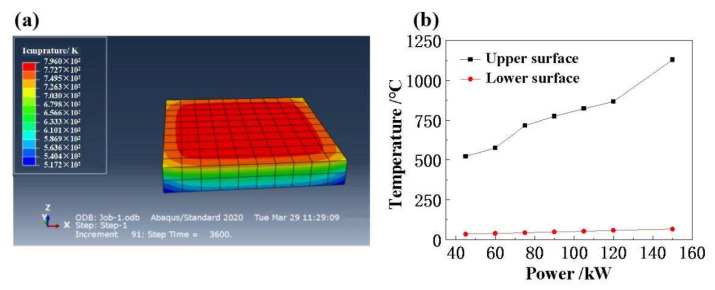
(**a**) Finite element simulation model of fused quartz ceramics. (**b**) Finite element simulation results of upper and lower surface temperature of quartz array with different input powers.

In this paper, choosing to use simulation to study some thermal conductivity of the material can significantly reduce the costs related to experiments, and can also provide stronger theoretical guidance for the preparation and practical application of materials. Therefore, we performed simulations before the experiments. The heat transfer module is analyzed using Abaqus element software. From the current literature of finite element analysis of heat flow sensors, Abaqus has more prominent advantages in nonlinear calculation, and in the software we simulate the density of heat flow by adding a boundary heat source. The sample size was set to 100 mm × 100 mm × 15 mm, the material characteristic parameters are shown in Table 1 and the initial environmental temperature is 20 °C. The three-dimensional temperature distribution of the concrete model structure of molten quartz ceramics is shown in Figure 2a, in which it can be found that the top layer of the material is at a higher temperature and the bottom layer is at a lower temperature, which can effectively protect it from the influence of the subsequent temperature increase in practical use. Therefore, in Figure 2b, we investigate the effect of quartz lamp arrays with different input powers on the temperature distribution at the top and bottom of the sample. From the calculation results of Abaqus finite element software, it was suggested that the upper and lower surface temperature of the sample increased with the increase in quartz lamp control power. When the input power is 45, 60, 75, 90, 105, 120 and 150 kW, the upper surface temperature of the sample is 522.85, 574.54, 715.25, 773.85, 822.85, 866.85 and 1129.85 °C. However, the highest lower surface temperature of the sample is 65.30 °C.

## 3. Results and Discussion

**Figure 3:**
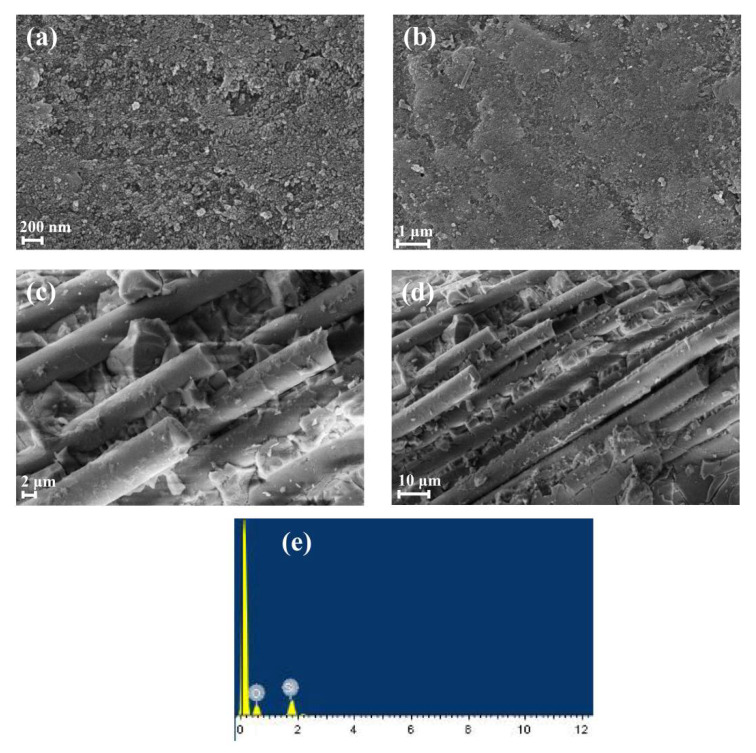
Scanning electron micrographs of samples at different scales: (**a**) 200 nm, (**b**) 1 μm, (**c**) 2 μm and (**d**) 10 μm. (**e**) Energy spectrum analysis of experimental samples.

In this study, we have obtained detailed information on the microstructure of the samples by scanning electron microscopy (SEM). As shown in Figure 3, we obtained SEM images of the samples at different positions (200 nm, 1 μm, 2 μm and 10 μm). As shown in Figure 3a–d, the surface of the sample shows dense and flat features, with an evenly distributed internal fiber skeleton and fiber diameter of about several microns. In addition, we found that the cracks around the fiber skeleton were filled with a large amount of quartz, which indicated that the quartz content of the sample was very high. In addition, as shown in Figure 3e, an energy dispersive spectrometer (EDS) was used in combination with SEM for analysis. From the results of the EDS test, we concluded that the sample contained 100% of both Si and O. In addition, we also carried out X-ray fluorescence spectroscopy and the results showed that the main component of the sample was SiO_2_, accounting for 96.95% of the total composition. The remaining part was mainly composed of a mixture of some oxides, including ferric oxide, alumina and phosphorus pentoxide. 

**Figure 4:**
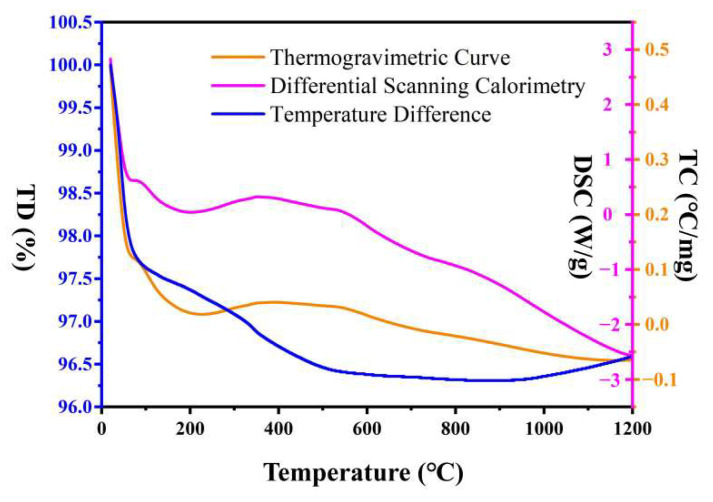
Sample DSC-TD-TC analysis profiles.

Dynamic thermal analysis is important for the study of the thermal properties and thermal stability of materials. Therefore, as shown in Figure 4, we studied the results of dynamic thermal effect analysis for samples up to 1200 °C. During the dynamic thermal effect test, the total weight loss of the sample was about 3.9625%. This is due to the low volatile matter content of the sample after the high-temperature combustion [33,34,35]. The general trends in the thermogravimetric curve (TC) can be divided into three obvious loss intervals. The first significant loss interval is between 25 and 200 °C. The weight loss rate is 2.568%. This weight loss is caused by the desorption of the adsorbed water and decomposition of the material; such weight loss is usually consistent with the temperature range of the first weight loss peak in differential thermogravimetry (DTG) [36]. This shows that desorption of adsorbed water and decomposition of the material occur in this temperature range, which is a natural process when samples are exposed to high temperatures. The second significant weight loss occurred in the temperature range of 200 to 800 °C. The weight loss rate was about 1.118%. According to the differential scanning calorimetry (DSC) curve, there is a small endothermic effect at 352.21 °C. This weight loss can be attributed to a phase transition or crystal transition of the sample. This indicates that the sample has undergone a phase transition or crystal transition reaction within this temperature range. The third significant weight loss occurred in the temperature range between 850 °C and 1200 °C. The mass of the substance increased. The DSC curve continued to decrease. No obvious exothermic peaks were observed. This shows that some complex reactions have occurred in this temperature range, which leads to an increase in the sample quality.

**Figure 5:**
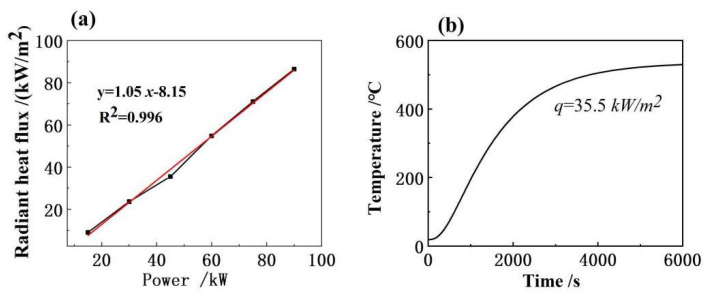
(**a**) Sample surface heat flux variation when the input power is 15, 30, 45, 60, 75 and 90 kW. (**b**) Curve of upper surface’s temperature showing that the heat flow density is 35.5 kW/m^2^.

We used a proprietary ALHS heat flux sensor to measure the surface heat flux density of the sample at different quartz lamp input powers. This sensor has the advantage of high accuracy and sensitivity and is capable of accurately measuring the heat flux density of the sample surface. Through the analysis of experimental data, it is suggested that the density of the radiant heat flux density gradually increases with the increase in input power. When the quartz lamp input power is 15, 30, 45, 60, 75 and 90 kW, the surface heat flow is measured as 9.05, 23.6, 35.5, 54.8, 71.1 and 86.5 kW/m^2^. This is in line with our expectations. Figure 5a shows that the power of the quartz lamp has a good linear relationship with the radiant heat flux density, with a correlation coefficient of 0.996. This demonstrates the high reliability and accuracy of our experimental data. Figure 5b shows the curve of the upper surface’s temperature and that the heat flux density is 35.5 kW/m^2^. It suggests that when the heat flux density of the sample surface is 35.5 kW/m^2^, as time increases, the upper surface temperature of the sample gradually increases, reaching a maximum temperature of 529.9 °C. This is similar to 522.85 °C, the result of the simulation. This shows that our experimental data are consistent and comparable with the simulation results, and verifies the correctness of the simulation results. It can also be seen that the heating rate gradually decreases as time increases, which is related to the thermal conductivity and thermal diffusion coefficient of the materials [37,38,39]. Overall, the experimental and simulation results are in good agreement and our fiber-reinforced fused silica ceramics can provide strong support and assistance for the thermal protection of future supersonic aircraft.

**Figure 6:**
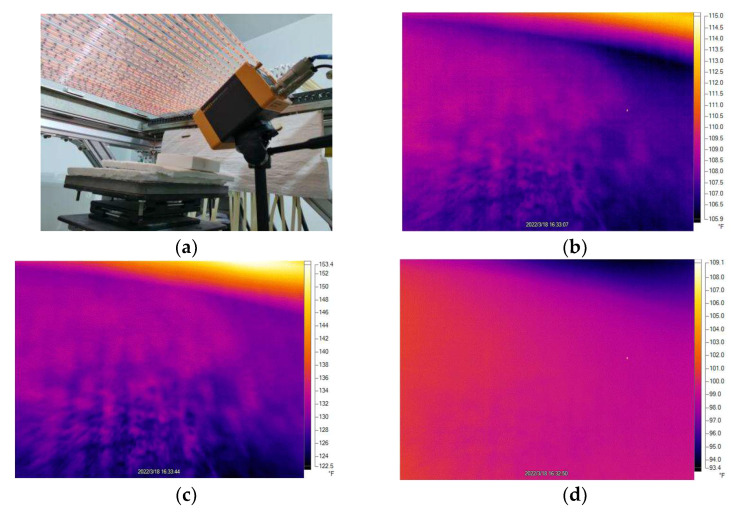
Results of the upper surface temperature uniformity test. (**a**) Photograph of the infrared camera temperature measuring device. (**b**–**d**) The surface temperature distribution of the measured sample at t = 0 s, 120 s and 240 s.

The ALHS heat flow sensor can only measure the temperature distribution on the lower surface of the material, whereas an infrared camera, as a high-precision temperature measuring tool, can monitor and analyze the surface temperature of samples in real-time. Therefore, in this experiment, we used an infrared camera to analyze the temperature uniformity of the sample, and the results obtained are shown in Figure 6a–d. As can be seen from the graph, the maximum temperature in the homogeneous surface region of the sample surface gradually increased as time increased, eventually reaching 95 °C. This shows that there is a certain temperature gradient in the heating process of the sample, and the temperature gradient increases gradually with the passage of time. Further research showed that the surface temperature of the sample is closely related to the distribution of fiber skeleton. Fiber-reinforced quartz glass is a kind of composite material that is mainly composed of a quartz glass matrix and fiber-reinforced materials. During the heating process, the heat transfer rate of the quartz glass matrix and fiber-reinforced materials is different, which leads to the uneven distribution of the surface temperature of the sample [40,41]. In contrast, the rod-shaped fiber backbone has a slow heat transfer rate, and it can easily form a hot zone at high temperatures. Therefore, the fiber skeleton of fiber-reinforced quartz glass has an important influence on its heat transfer performance. In order to further improve the heat transfer performance of fiber-reinforced quartz glass, some measures can be taken. For example, a more uniform fiber distribution can be used in the preparation process to reduce the effect of fiber skeleton on heat transfer. At the same time, the composition and structure of the quartz glass matrix can be optimized to improve its heat transfer properties.

**Figure 7:**
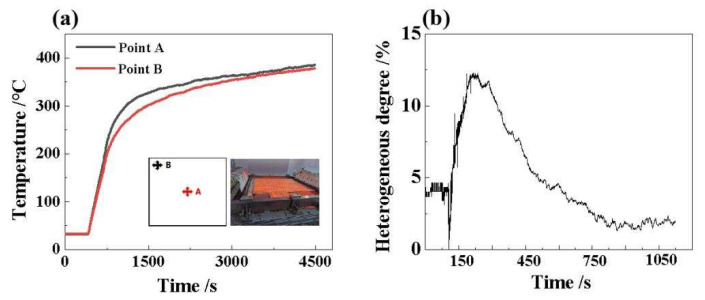
(**a**) Temperature change curves at different positions on the upper sample surface, (**b**) upper surface temperature inhomogeneity over time.

When studying the thermal properties of materials, the non-uniformity of radiation surface temperature is a very important parameter [42,43]. This parameter can be defined by the temperature difference in the irradiated surface temperature between two points; that is, it represents the average temperature of two points on the irradiated surface. In order to better explore the characteristics of the distribution of sample surface uniformity at high temperatures, a type k thermocouple was used to measure the temperature at different locations on the sample surface [44,45]. When the input power of quartz lamp is 1 kW, we measured the temperature changes at the center and the vertex angle, as shown in Figure 7a. The results show that the surface temperature of the sample presents a distribution pattern of high center and low surround, which is basically consistent with the results of finite element simulation and is also related to the Gaussian distribution of the light source spectrum [46,47,48]. As time progresses, the temperature at the center and the edge of the sample is basically the same, which indicates that the heat transfer process is close to becoming stable. Figure 7b shows that the overall trend in the uncertainty of the upper surface temperature over time increases and then decreases, with a maximum uncertainty of 12.28%. This result shows that when studying the thermal characteristics of a material, we need to consider the influence of time and space on the temperature distribution. In addition, we need to analyze and process the measurements results comprehensively to obtain more accurate data. In a word, this study provides an important reference for us to understand the thermal properties of materials in depth.

Table 2 shows the comparison between our designed material and other similar materials in terms of temperature inhomogeneity performance and thermal insulation efficiency [49,50,51]. As far as temperature inhomogeneity is concerned, our materials have a high degree of uniformity, which means that our materials are easier to check and repair in practice. This is due to the advanced manufacturing technology used in our materials, which results in a more uniform temperature distribution within the material, thus reducing failures and damage caused by temperature inhomogeneities. In addition, the fiber-reinforced Shi Ying glass we designed has a very strong thermal insulation effect. This means that our materials have good thermal insulation capacity, which can provide additional ideas and methods for developing various thermal insulation materials. This is due to the use of high-quality fiber-reinforced material, which allows the heat transfer performance of the material to be effectively controlled. Generally speaking, our materials are expected to be used in a wide range of applications because of their excellent performance in temperature inhomogeneity and insulation efficiency. We believe that with the progress of technology and the continuous improvement of material manufacturing technology, our materials will be more widely used and popularized in the future.

## 4. Conclusions

(1) The upper and lower surface temperatures of the sample display an increasing trend as the control power of the quartz lamp increases. When the input power is into 45, 60, 75, 90, 105, 120 and 150 kW, the upper surface temperature of the sample is 522.85, 574.54, 715.25, 773.85, 822.85, 866.85 and 1129.85 °C, respectively, while the highest lower surface temperature of the sample is 65.30 °C.
(2) The radiant heat flux density of quartz lamp array increases as the input power increases. When the input power of the quartz lamp is 15 kW, 30 kW, 45 kW, 60 kW, 75 kW and 90 kW, the surface radiant heat flux is measured as 9.05, 23.6, 35.5, 54.8, 71.1 and 86.5 kW/m^2^, respectively. There is a good linear relationship, with a correlation coefficient of 0.996.
(3) The fiber skeleton structure in fiber-reinforced fused quartz ceramics has a significant effect on the heat transfer performance, and the longitudinal heat transfer along the rod fiber skeleton is slower. The surface temperature distribution of the sample shows the distribution pattern of high center and low surround, and the maximum inhomogeneity of the upper surface temperature of the sample is 12.28%. However, as time progresses, the center and edge temperatures of the specimen tend to be basically uniform.

## Figures and Tables

**Table 1:** The material properties of the sample.

Thermal Conductivity W/(m·K)	Density kg/m^3^	Specific Heat Capacity J/(kg·K)	Emissivity	Initial Temperature/K
0.84	1.92	1100	0.75	292.15

**Table 2:** Comparison of the temperature inhomogeneity properties and insulation efficiency of our designed material with other similar materials.

References	[49]	[50]	[51]	This Work
Temperature inhomogeneity	35%	30%	-	12.28%
Heat insulation efficiency	-	-	83.7%	94.2%

## Data Availability

Publicly available datasets were analyzed in this study. These data can be found here: [https://www.lumerical.com/ (accessed on 1 January 2020)].
